# Analyzing myocardial torsion based on tissue phase mapping cardiovascular magnetic resonance

**DOI:** 10.1186/s12968-016-0234-5

**Published:** 2016-04-10

**Authors:** Teodora Chitiboi, Susanne Schnell, Jeremy Collins, James Carr, Varun Chowdhary, Amir Reza Honarmand, Anja Hennemuth, Lars Linsen, Horst K. Hahn, Michael Markl

**Affiliations:** Jacobs University Bremen, Bremen, Germany; Fraunhofer MEVIS, Bremen, Germany; Department of Radiology, Northwestern University, Chicago, IL USA

**Keywords:** Non-ischemic cardiomyopathy, Myocardial velocities, Torsion, Cardiovascular magnetic resonanace, Tissue phase mapping

## Abstract

**Background:**

The purpose of this work is to analyze differences in left ventricular torsion between volunteers and patients with non-ischemic cardiomyopathy based on tissue phase mapping (TPM) cardiovascular magnetic resonance (CMR).

**Methods:**

TPM was performed on 27 patients with non-ischemic cardiomyopathy and 14 normal volunteers. Patients underwent a standard CMR including late gadolinium enhancement (LGE) for the assessment of myocardial scar and ECG-gated cine CMR for global cardiac function. TPM was acquired in short-axis orientation at base, mid, and apex for all subjects. After evaluation by experienced observers, the patients were divided in subgroups according to the presence or absence of LGE (LGE+/LGE-), local wall motion abnormalities (WM+/WM-), and having a preserved (≥50 %) or reduced (<50 %) ejection fraction (EF+/EF-). TPM data was semi-automatically segmented and global LV torsion was computed for each cardiac time frame for endocardial and epicardial layers, and for the entire myocardium.

**Results:**

Maximum myocardial torsion was significantly lower for patients with reduced EF compared to controls (0.21 ± 0.15°/mm vs. 0.36 ± 0.11°/mm, *p* = 0.018), but also for patients with wall motion abnormalities (0.21 ± 0.13°/mm vs. 0.36 ± 0.11°/mm, *p* = 0.004). Global myocardial torsion showed a positive correlation (*r* = 0.54, *p* < 0.001) with EF. Moreover, endocardial torsion was significantly higher than epicardial torsion for EF+ subjects (0.56 ± 0.33°/mm vs. 0.34 ± 0.18°/mm, *p* = 0.039) and for volunteers (0.46 ± 0.16°/mm vs. 0.30 ± 0.09°/mm, *p* = 0.004). The difference in maximum torsion between endo- and epicardial layers was positively correlated with EF (*r* = 0.47, *p* = 0.002) and age (*r* = 0.37, *p* = 0.016) for all subjects.

**Conclusions:**

TPM can be used to detect significant differences in LV torsion in patients with reduced EF and in the presence of local wall motion abnormalities. We were able to quantify torsion differences between the endocardium and epicardium, which vary between patient subgroups and are correlated to age and EF.

## Background

Left ventricle (LV) torsion, the counter-rotation of basal versus apical regions, is known to play a crucial role in cardiac contraction and relaxation [1, 2]. The kinematics of the microfibers having different orientation in the myocardial wall enables rapid blood ejection and refilling during the cardiac cycle. Studies have shown that changes in the rotation patterns of the heart muscle and specifically LV torsion are connected to various cardiac diseases [3] such as aortic valve stenosis [4], ischemia [5, 6], or dilated and hypertrophic cardiomyopathy [7].

Previous studies have attempted to quantify myocardial torsion using tissue Doppler imaging [8], or speckle tracing echocardiography [9]. However, Doppler imaging cannot measure velocity in three dimensions and for all cardiac segments. Speckle tracking, while overcoming the previous limitations, is highly dependent on the acoustic window. Cardiovascular magnetic resonance (CMR) is a powerful alternative for analyzing local myocardial deformation. Based on MR-Tagging [10, 11], the rapid and complex rotational motion pattern and the endo- versus epicardial differences of the heart muscle are difficult to quantify due to the low spatial resolution. Displacement-encoded image using stimulated echoes (DENSE) [12] and Tissue Phase Mapping (TPM) [13] can measure local motion with higher temporal (DENSE) and spatial (TPM) resolution respectively. Moreover, TPM directly measures myocardial velocities and allows for their quantitative assessment along all three principal motion directions of the heart (radial, long-axis, circumferential). Acceleration plays an important role to make high resolution TPM acquisition possible during breath hold. Existing methods employ spatio-temporal imaging acceleration k-t GRAPPA [14] (required breath hold: 25 heartbeats) and non-Cartesian SENSE implemented on the GPU [15] (required breath hold: 13–17 heartbeats).

Previous studies have shown the potential of TPM to detect disturbed regional myocardial motion in patients with hypertrophy [16], cardiomyopathy [17], after heart transplantation [18], and other cardiac diseases [3–7]. Moreover, it has been demonstrated that TPM can be employed to assess the temporal dynamics of rotational velocities during the cardiac cycle. By comparing basal versus apical LV rotation velocity, the twisting and untwisting pattern of the heart could be evaluated [19]. Previous TPM studies [16–18] have measured myocardial torsion, but a systematic quantification of endocardial and epicardial torsion and analysis of the variability of torsion-related parameters across patient cohorts has not been performed to date.

Myocardial twist has also been previously studied using local shear, mainly based on tagged CMR [20]. The local measures for myocardial twist are circumferential-radial and circumferential-longitudinal shear which are derived based on the relative displacement and reported using the American Heart Association (AHA) model. Even though local twist measures exist, it is possible that when the rotational velocity is only locally affected, this may also have a significant impact on the global LV twist due to the complex fiber structure. The goal of this work is to investigate a global parameter that can measure the total LV torsion during the cardiac cycle. Using a single, global measure, we ultimately aim to detect changes in the LV mechanics before they impact the ejection fraction or even manifest as visible wall motion abnormalities in cine CMR. Based on a simplified mechanical model, the proposed global torsion takes into account the LV radius and length, measuring the total relative rotation of the basal myocardium with respect to the apex.

We present a method to quantify the myocardial and transmural (epicardial and endocardial) torsion using TPM imaging followed by a semi-automatic processing pipeline in a study cohort of 27 patients with non-ischemic cardiomyopathy and 14 normal controls. Our goal was to test the hypothesis that TPM can by employed to detect significant differences in LV torsion in patients with reduced ejection fraction (EF) and wall motion abnormalities (WM) compared to patients with preserved EF and control subjects.

## Methods

### Study cohort

The study cohort included 27 patients (15 females, age = 50 ± 18 years) with non-ischemic cardiomyopathy (NICM) in normal sinus rhythm and 14 healthy control subjects (5 females, age 49 ± 18 years), as described in Table 1. Patients with primary or secondary causes of NICM were included; patients with a history of treated coronary artery disease without residual obstructive lesions in whom LV dysfunction was out of proportion to the extent of treated coronary artery disease were considered to have an NICM and were also included. The patient cohort consisted of: *n* = 10 subjects with restrictive cardiomyopathy, *n* = 9 subjects with dilated cardiomyopathy, *n* = 3 subjects with hypertrophic cardiomyopathy, *n* = 3 subjects with inflammatory cardiomyopathy, and *n* = 2 subjects with non-classified cardiomyopathy. All study subjects were included in accordance with the Institutional Review Board (IRB) of Northwestern University protocol, which permitted retrospective chart review in patients. Volunteers were recruited under an IRB approved prospective protocol and informed consent was obtained from all participants.

**Table 1:** Volunteers and patients groups and subgroups

	All Patients	Volunteers	EF+	EF-	WM+	WM-	LGE+	LGE-
Number	27	14	15	12	17	10	17	10
Age	50 ± 18 (18–77)	49 ± 18 (20–75)	52.9 ± 17.4 (25,77)	46.3 ± 17.1 (18,72)	42.8 ± 16.4 (18,72)	63 ± 10.7 (39,77)	54.3 ± 17.4 (18,77)	43.4 ± 15.4 (26,71)
Females	15	4	5	10	9	6	9	6
EF	47.4 ± 17.1 (15–66)	64.7 ± 8.1 (49–79)	60.1 ± 5.1 (50,66)	32.5 ± 11.3 (13,46.2)	39 ± 16.2 (13,65)	61.7 ± 3.8 (52,66)	47 ± 18.6 (13,66)	48.1 ± 14 (21,64.5)

Statistics for patients with preserved ejection fraction (EF+), and reduced ejection fraction (EF-) respectively, patients with wall motion abnormalities (WM+), without wall motion abnormalities (WM-) respectively, patients with late gadolinium enhancement (LGE+), and patients without late gadolinium enhancement (LGE-)

### MR imaging

All patients underwent standard-of-care CMR using 1.5 T MR systems (Aera and Avanto, Siemens Medical Systems, Erlangen, Germany). The examination included late gadolinium enhancement (LGE) for the assessment of myocardial scar as well as ECG gated time-resolved (cine) CMR for the evaluation of global cardiac function. In addition, patients underwent 2D TPM at basal, mid-ventricular (mid), and apical locations. TPM consisted of a black-blood prepared cine phase-contrast sequence with three-directional velocity encoding of myocardial motion (venc = 25 cm/s, temporal resolution = 20.8–22.4 ms, spatial resolution = 2.0–2.4 mm x 2.0–2.4 mm × 8 mm). Spatio-temporal imaging acceleration (k-t GRAPPA) with a net acceleration factor of R_net_ = 3.6 was employed, which permitted data acquisition during breath-holding. The breath-hold time was 25 heart beats per slice. In all control subjects, a repeat CMR exam (rescan) including TPM was performed two weeks later to assess scan-rescan reproducibility of TPM parameters.

### Data analysis - LGE and cine CMR

**Fig. 1:**
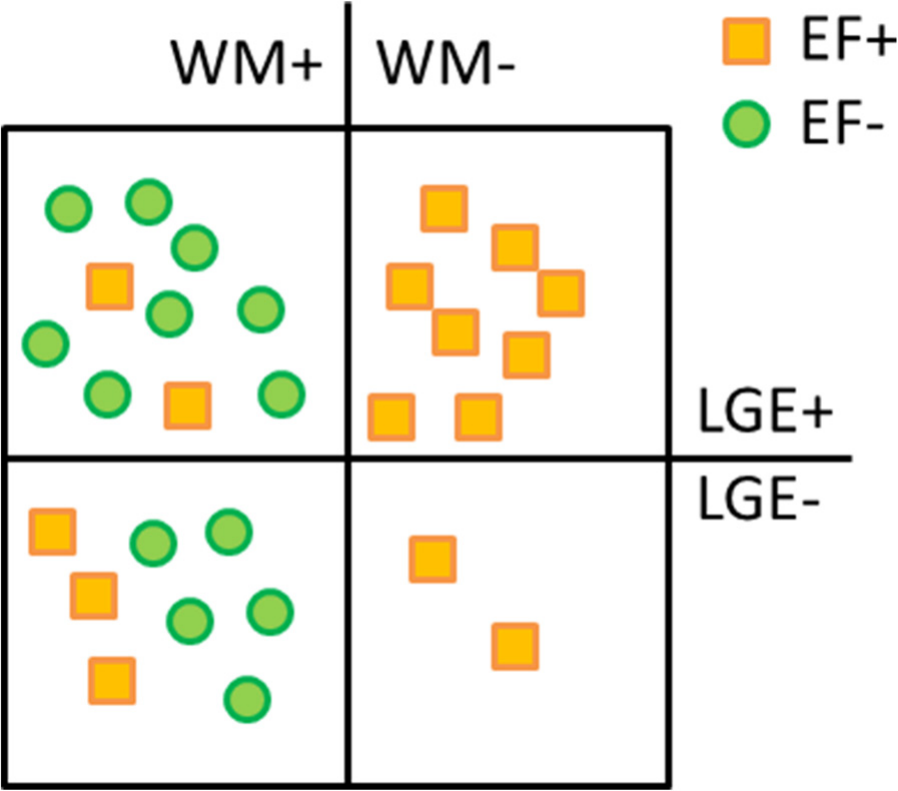
Patients categorization by ejection fraction (EF+ yellow, EF- green markers), by wall motion abnormalities (WM + left, WM- right), and by the presence of late gadolinium enhancement (LGE+ top, LGE- bottom)

LGE and cardiac cine CMR were qualitatively evaluated by an experienced observer based on the AHA 16-segment model [21]. LGE analysis included the assessment of presence of late gadolinium enhancement. Patients were accordingly divided into two subgroups with (LGE+) and without late gadolinium enhancement (LGE-), without further considering the size or the extent of the enhanced region. Regional wall motion was classified according to the following scheme: Normal wall motion (0), mildly hypokinetic (1), moderately hypokinetic (2), severely hypokinetic (3), akinetic (4), or dyskinetic (5). Patients were assigned to the abnormal wall motion subgroup (WM+) if at least one segment was graded >1, and to normal wall motion (WM-) otherwise. Similarly, patients were grouped according to the EF < 50 % in two subgroups with reduced (EF-) and preserved (EF+) heart function. The patients’ distribution in subgroups, as well as the subgroup overlap is shown in Fig. 1.

### TPM data analysis and torsion quantification

TPM data were analyzed using a customized software solution built using the MevisLab platform [22]. Pre-processing included correction for eddy currents using a first-order linear approximation [23]. Static soft tissue with low standard deviation in all three velocity directions was semi-automatically located. First, the user drew a rectangular region of interest around the heart. The soft tissue regions were then determined by classifying the histogram of the magnitude image using a Gaussian mixture model with three classes for background, soft tissue, and solid tissue. The soft tissue image region was filtered by the standard deviation (SD) in each velocity direction (v_x_,v_y_,v_y_) over the temporal series. Pixels with SD(v_x_) < 5 mm/s, SD(v_y_) < 5 mm/s and SD(v_z_) < 5 mm/s were considered stationary. A linear fit was performed for each velocity component of the resulting stationary tissue, which was subtracted from the velocity field. To reduce the noise level, a low-pass 3x3 SD filter was applied.

**Fig. 2:**
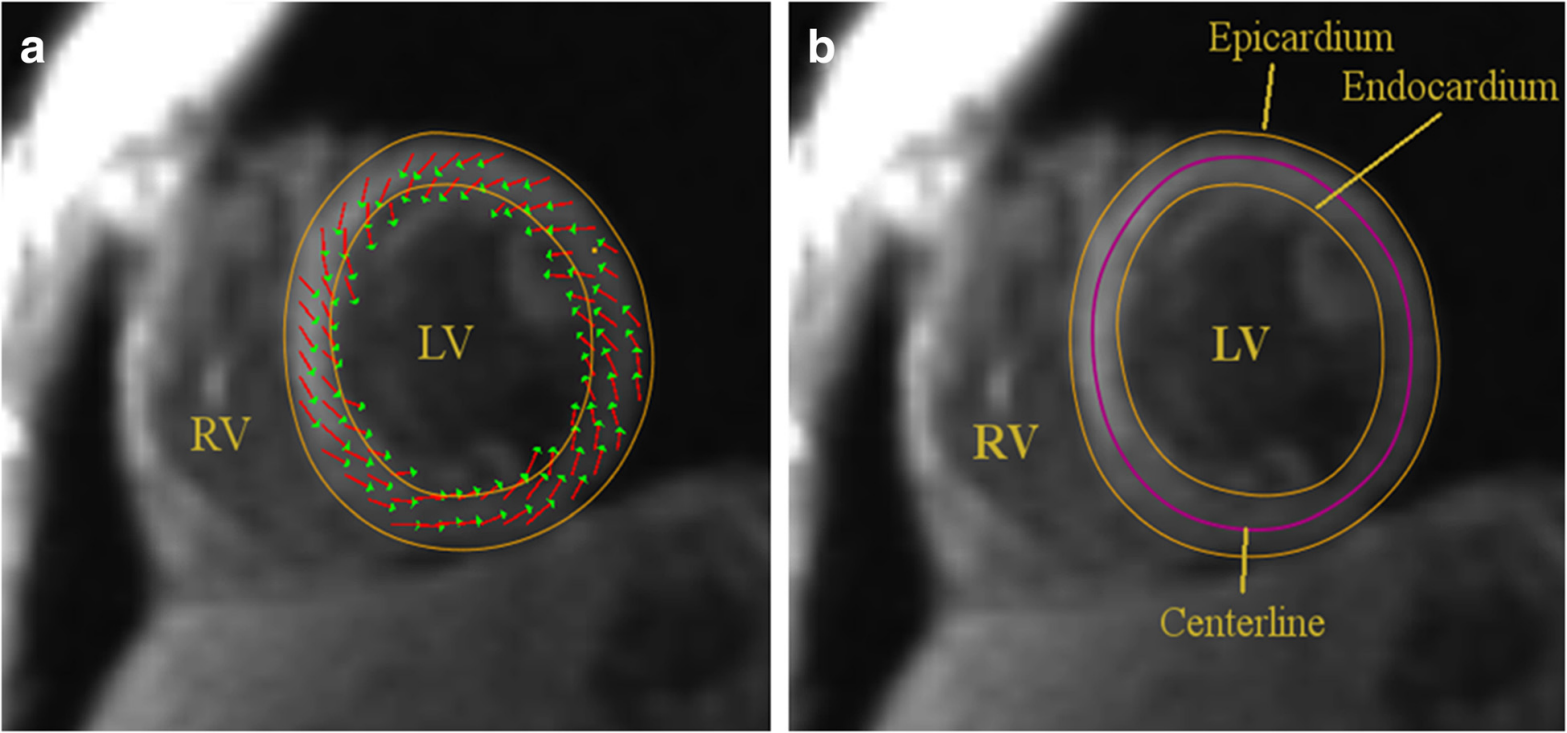
**a** Myocardial contours enclosing the local myocardial velocity field (planar components) for base slice in early systolic phase. **b** Automatic epicardium and endocardium contours (yellow), and centerline (purple) at the same slice location. Endocardial region spans between endocardium and centerline, and epicardial region between epicardium and centerline, respectively

Semi-automatic myocardium segmentation was subsequently performed. Two manual epi- and endocardial contours were drawn for each slice in one time point in the middle of the cardiac temporal sequence during systole. The manual contours initialized an active contour model, using the in-plane projected velocity field as guiding forces (Fig. 2-a). The contours were then propagated over the entire image series using an approach similar to [24]. During contour propagation, the Runge-Kutta method was used to integrate the velocity field over time and determine the local myocardial displacement in each time frame. Finally, morphological closing [25] was applied to the resulting myocardial mask to smooth the border. The semi-automatic segmentation algorithm was presented and evaluated in [26].

In order to distinguish between endocardial and epicardial regions, the centerline of the myocardial mask was automatically extracted using a skeletonization approach based on topology-preserving morphological thinning by Selle et al. [27]. The voxels delimited by the endocardial contour and the centerline were labeled as endocardial myocardium and voxels between centerline and epicardium contours were labeled as epicardial (Fig. 2-b).

To remove translational motion of the whole heart from the velocity fields, a correction based on subtraction of global translation velocities in X and Y directions from the local velocity components was performed based on a previously reported strategy [28]. The three-directional LV velocities (v_x_,v_y_,v_z_) were transformed into a cylindrical coordinate system (v_r_,v_ϕ_ ,v_z_) where the base of the cylinder was parallel to the short-axis slice and the center was at the center of the myocardium mask. The resulting velocity components were thus radial v_r_ (positive-contraction/negative-expansion), long-axis v_z_ (positive-shortening/negative-lengthening), and rotational v_ϕ_ (positive-clockwise/negative-anticlockwise) as previously described in [13].

The average rotational velocity of each myocardial slice was divided by the average myocardium radius *R* for every time point, in order to obtain the angular velocity:

$$ {\omega}_t=\frac{v_{\phi }}{R} $$

**Fig. 3:**
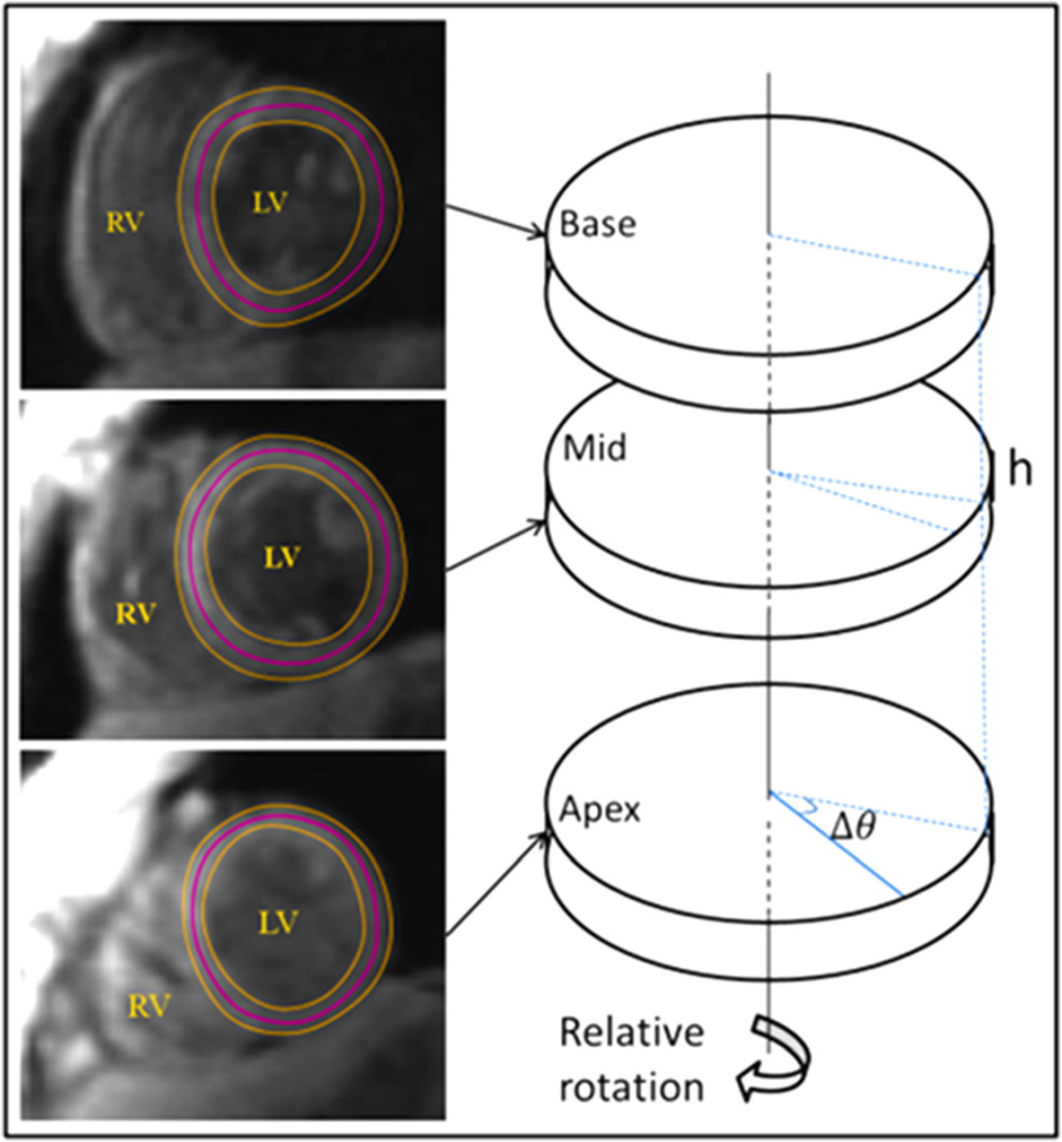
Rotational velocity from TPM for base, mid, and apical slices determines the relative rotation angle ∆θ between the three LV slices

As shown in Fig. 3, the rotation angle *θ*_*t*_ was computed by integrating the angular velocity over time for the base, mid, and apical slices. The myocardial torsion *T* between base and apex at time *t* during the cardiac cycle was defined as their relative rotation angle *Δθ*_*t*_ normalized by the distance *h* between the respective slices:

1 $$ {T}_t^{AB}=\frac{\varDelta {\theta}_t}{h} $$

The distance between slices *h* is assumed to be fixed, i.e. the long axis shortening of the LV was not taken into account. Out assumption is based on the fact that the position of the basal and apical imaging planes remains static during acquisition. In general, using Eq. 1, the myocardial torsion can be computed for any pair of short-axis slices: base-apex, base-mid, or mid-apex.

Torsion was computed for the whole myocardium, as well as for the endocardial and epicardial regions, respectively. In addition, maximum torsion for the entire myocardium *T*_*max*_ during the cardiac cycle was quantified. We assumed that the maximum of the torsion curve for the global myocardium, as well as for epi- and endocardial regions would be reached during end-systole. Absolute and relative difference in magnitude between maximum endocardial and epicardial torsion were also calculated:

2 $$ \begin{array}{l}\varDelta {T}_{max}={T}_{max}^{Endo}-{T}_{max}^{Epi}, \kern0.62em \mathrm{and}\hfill \\ {}\varDelta \%{T}_{max}=\frac{T_{max}^{Endo}-{T}_{max}^{Epi}}{T_{max}^{Endo}}\hfill \end{array} $$

To compare the temporal evolution of LV torsion over time for different groups, torsion-time curves for volunteers and patients with reduced and preserved EF were resampled at 5 ms time steps and then averaged.

To assess inter-observer variability, TPM torsion analysis was performed by two independent observers, blinded to all subject information. The reproducibility of the entire analysis was tested on the 14 volunteers, who were scanned a second time. The semi-automatic segmentation was performed by the same observer.

### Statistics

All continuous parameters are reported as mean ± standard deviation. Differences in parameters between subject groups were assessed using the standardized t-test (two-sample, unequal variance) for normal distributions and Wilcoxon rank sum test otherwise [29]. We computed Pearson’s correlation coefficient R to assess relationships between torsion, age, and EF, a correlation with *p* < 0.05 was considered significant. Inter-observer variability and scan-rescan reproducibility were analyzed using Bland-Altman plots and by calculating mean differences and limits of agreement (mean ± 1.96xSD of the differences).

## Results

TPM data from all subjects was successfully processed with our method. The processing time per subject was 10–20 min, depending on heart rate and thus number of time cardiac frames.

### Study cohort

Our study cohort consisting of 27 patients and 14 volunteers is described in more detail in Table 1. The groups of patients and volunteers had similar age ranges with an average age of 50 ± 18 for patients and 49 ± 18 for volunteers. Of the 27 patients, 12 had a reduced EF while for 15 the EF was preserved. The subgroup of patients with reduced EF (EF-) had an average EF of 32.5 ± 11.3 compare to 60.1 ± 5.1 for the subgroup with preserved EF (EF+). Also, for 17 of the patients the wall motion was impaired, while for the other 10 it was normal. The subgroup of patients with wall motion abnormalities WM+ had an average EF of 39 ± 16.2 compared to 61.7 ± 3.8 for the patients without wall motion abnormalities WM-. Out of the WM+ subgroup, 5 of the patients had a preserved EF, while the other 12 had a reduced EF. Also, for a subset of 17 of the total number of patients late gadolinium enhancement was present (LGE+), while for the rest of 10 there was no enhancement (LGE-). More detailed information about the overlap of the defined subgroups is given in Fig. 1.

### Global LV torsion

**Fig. 4:**
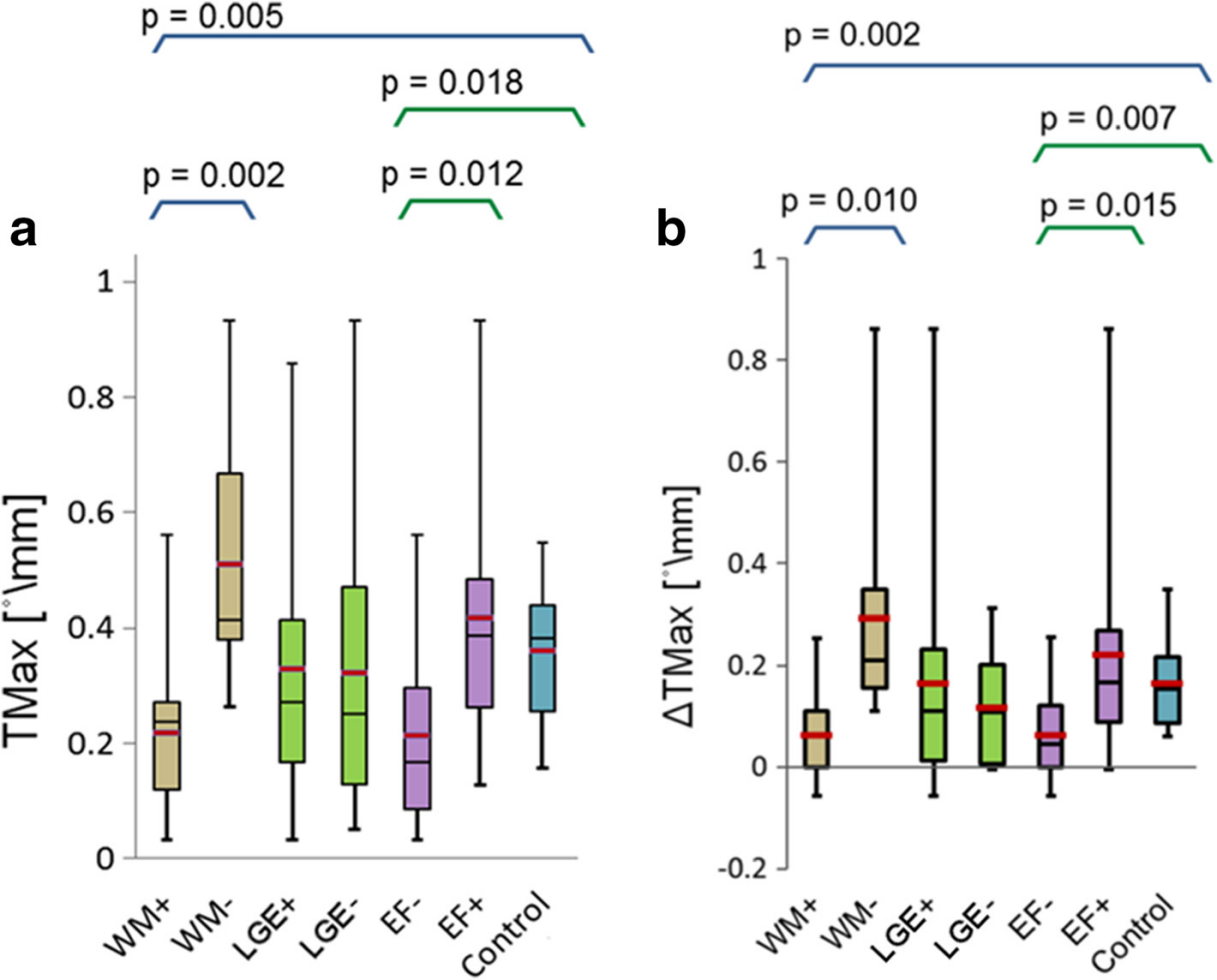
**a** - Maximum torsion for patient subgroups WM+/−, LGE+/−, EF+/−, and healthy controls. **b** - Maximum torsion for patient subgroups: with and without wall motion abnormalities (WM+/−); with reduced (EF-) and preserved (EF+) global function in green; and healthy controls in orange

When comparing the subgroups EF+ and EF-, we found a significantly lower myocardial *T*_*max*_ for the patients with reduced EF (EF-) compared to the EF+ group (0.21 ± 0.15°/mm vs. 0.41 ± 0.21°/mm, *p* = 0.012) and compared to the control group (0.21 ± 0.15°/mm vs. 0.36 ± 0.11°/mm, *p* = 0.018). Also, *T*_*max*_was significantly reduced for the group WM+ compared to WM- and the controls (0.21 ± 0.13°/mm vs. 0.51 ± 0.20°/mm, *p* = 0.002, and vs. 0.36 ± 0.11°/mm, *p* = 0.004), as shown in Fig. 4-a. We also separately considered only the 5 patients from the WM+ group who had a preserved EF. Their average *T*_*max*_ was 0.23 ± 0.05°/mm, which amounts to only 63.8 % or the average maximum torsion of the control group. However, when comparing patients with and without late gadolinium enhancement we found no significant difference in maximum torsion.

**Fig. 5:**
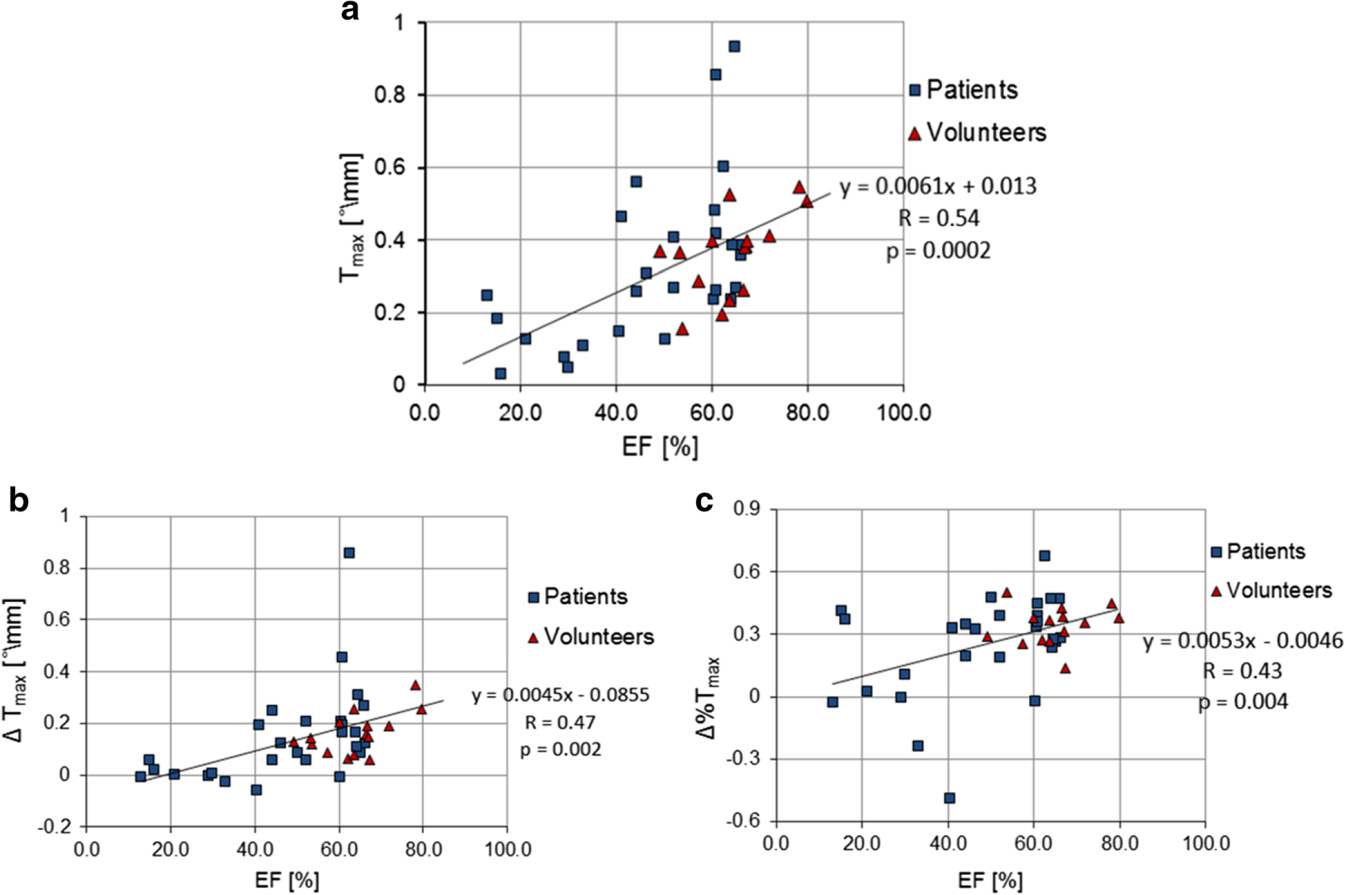
Torsion correlated with EF: (**a**) T_max_ over EF, (**b**) ∆T_max_ over EF, and (**c**) ∆%T_max_ over EF for patients with preserved and reduced EF and control subjects with normal EF. Trends for all subjects represented in black. Trendlines, R, and *p* values on the right

**Fig. 6:**
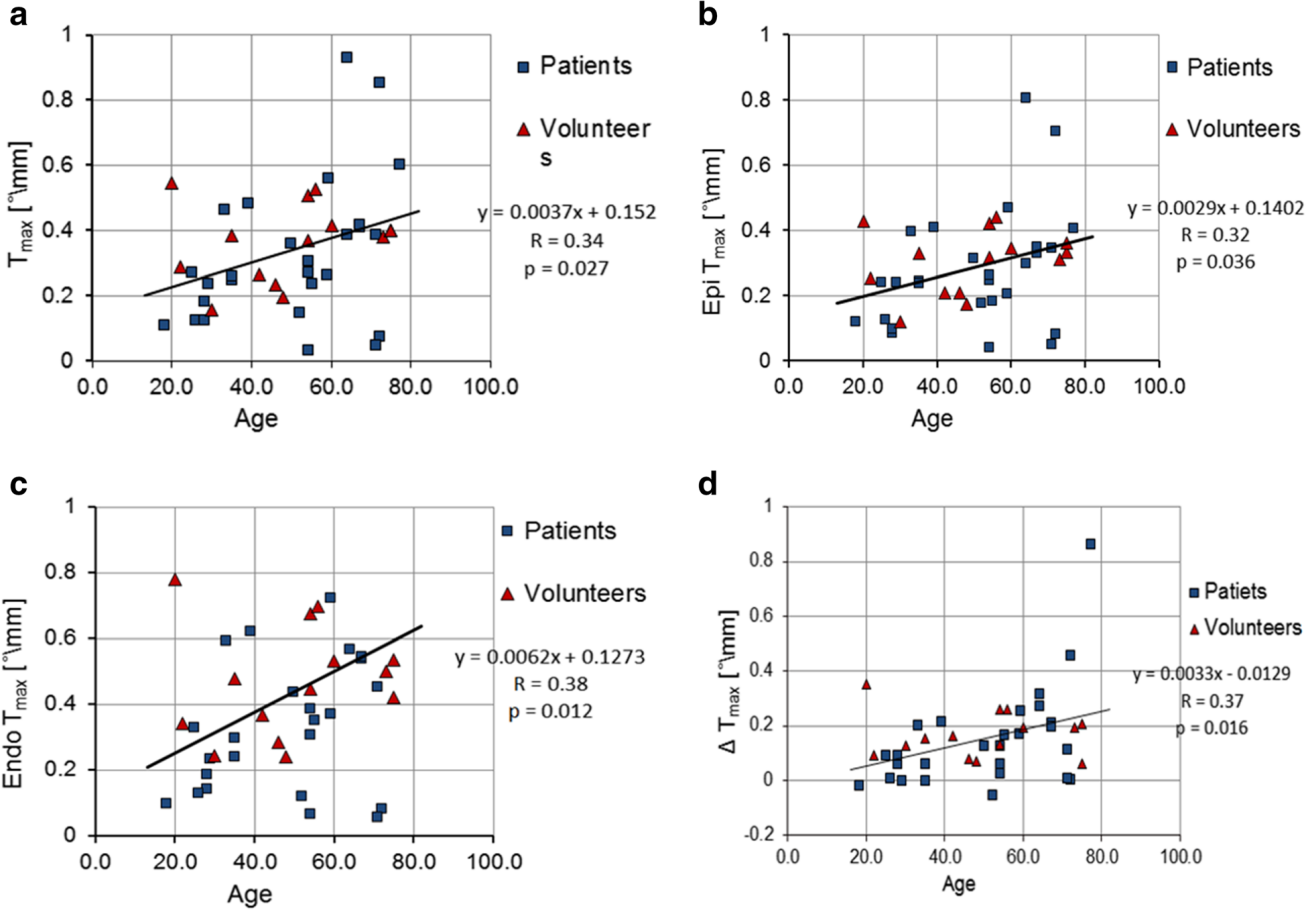
Torsion correlated with age: (**a**) *T*_*max*_ over age, (**b**) Epicardial *T*_*max*_ over age, (**c**) Endocardial *T*_*max*_ over age, and (**d**) ∆*T*_*max*_ over age for patients with preserved and reduced EF and control subjects with normal EF. Trends for all subjects represented in black. Trendlines, R and *p* values on the right

We found a strong positive correlation between *T*_*max*_ and EF (*r* = 0.54, *p* < 0.001) shown in Fig. 5-a, which indicates that reduced global cardiac function can negatively impact LV torsion. Additionally, we found a significant positive correlation between *T*_*max*_ and age (*r* = 0.34, *p* = 0.027), which was also present separately in the epicardial and endocardial bands (*r* = 0.32, *p* = 0.036 and *r* = 0.38, *p* = 0.012 respectively), as shown in Fig. 6-a,b,c. However, the correlation was not significant when considering only the small subgroup of volunteers (*p* = 0.4).

### Endocardial vs. epicardial torsion

The difference between endocardial and epicardial torsion ∆*T*_*max*_ were significantly reduced for patients with reduced EF (EF-) when compared to patients with preserved EF (EF+) and controls (0.06 ± 0.08°/mm vs. 0.22 ± 0.20°/mm, *p* = 0.015, and vs. 0.16 ± 0.08°/mm, *p* = 0.007). Similarly, patients with wall motion abnormalities WM+ had significantly lower transmural torsion difference ∆*T*_*max*_, compared to the controls and patients without wall motion abnormalities WM- (0.06 ± 0.08°/mm vs. 0.29 ± 0.21°/mm, *p* = 0.010, and vs. 0.16 ± 0.08°/mm, *p* = 0.002), as shown in Fig. 4-b. Also, when considering only those patients from the WM+ group with a preserved EF, the average ∆*T*_*max*_ amounted to 0.08 ± 0.05°/mm, which is only 50 % of the average torsion difference for the control group.

In Fig. 5-b,c, we analyzed the correlation between the difference between epicardial and endocardial torsion and EF. We found significantly positive associations between EF and both ∆*T*_*max*_ (*r* = 0.47, *p* = 0.002) and ∆%*T*_*max*_ (*r* = 0.43, *p* = 0.004). Additionally we observed a significant increase of ∆*T*_*max*_ (*r* = 0.37, p = 0.016) with age, as shown in Fig. 6-d when considering the entire subject cohort (no significant correlation was found for the subgroup of volunteers individually).

### Torsion-time curves

**Fig. 7:**
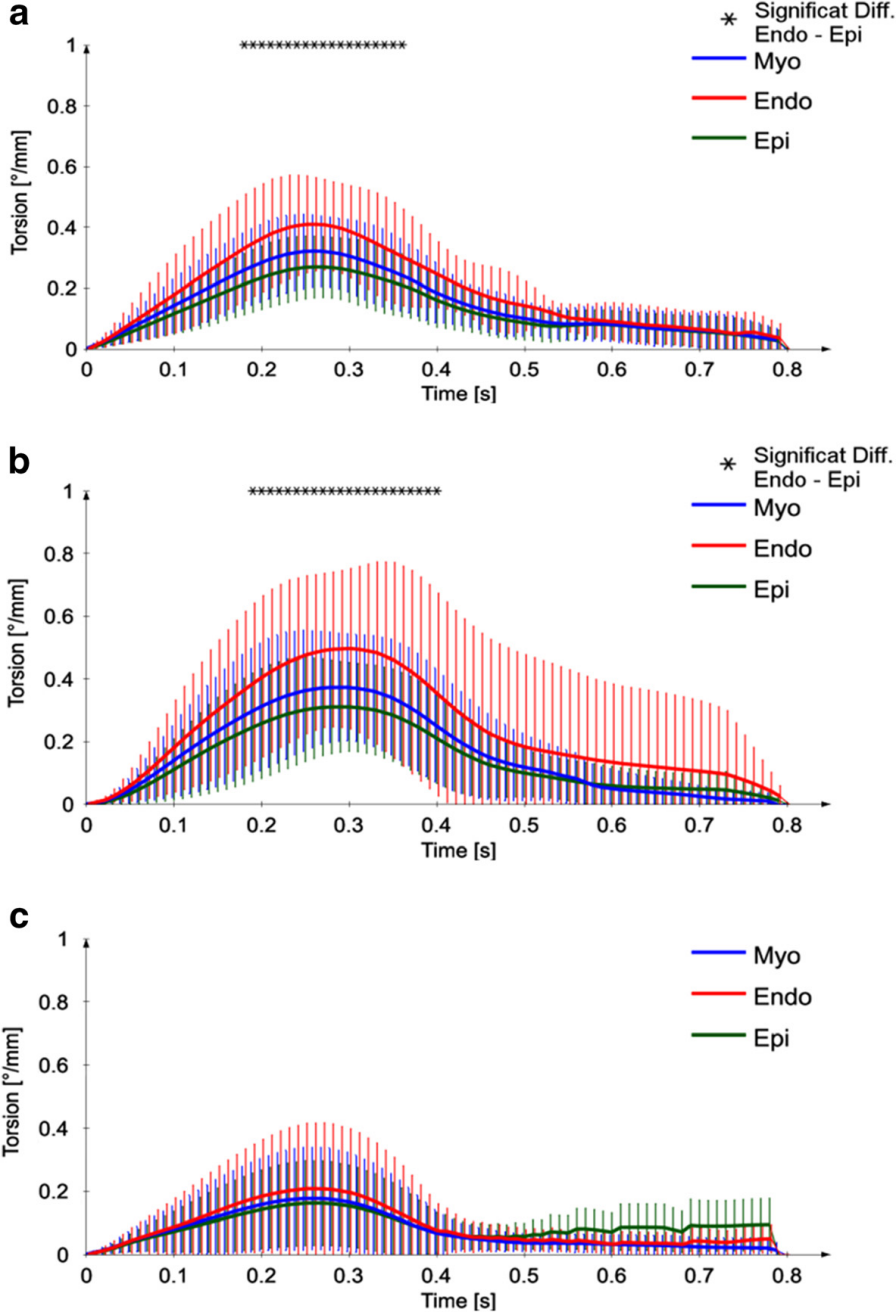
Average myocardial, endocardial, and epicardial curves for volunteers (**a**), patients with preserved (**b**), and reduced EF (**c**) over one cardiac cycle. The error bars represent the standard deviation across subject group. Time points were a significant difference (*p* < 0.05) between epicardial and endocardial torsion was observed are marked on the top with an asterisk (*)

Figure 7 shows the evolution of myocardial torsion as a function of time in the cardiac cycle for volunteers (A) and the patient subgroups with preserved (B) and reduced EF (C). Each graph shows full myocardial (blue) as well as endocardial (red), and epicardial (green) torsion. In the case of volunteers and for patients with preserved EF, endocardial torsion was significantly higher than the epicardial torsion throughout systole. The respective time points where this difference is significant are marked with (*) in Fig. 7. However, this difference was not observed for the patients with reduced EF.

The torsion curves reach a maximum during the end-systolic phase when the LV was fully contracted. The average maximum endocardial torsion was significantly larger than the epicardial torsion for both volunteers (0.46 ± 0.16°/mm vs. 0.30 ± 0.09°/mm, *p* = 0.004) and patients with preserved ejection fraction EF+ (0.56 ± 0.33°/mm vs. 0.34 ± 0.18°/mm, *p* = 0.039). Peak torsion was reached by the epi- and endocardial torsion curves concomitantly, i.e. we measured no statistically significant temporal delay. For this reason, when computing the difference between endo- and epicardial maximum torsion, we simply subtracted the absolute maxima of each curve, as previously defined in Eq. 2.

**Fig. 8:**
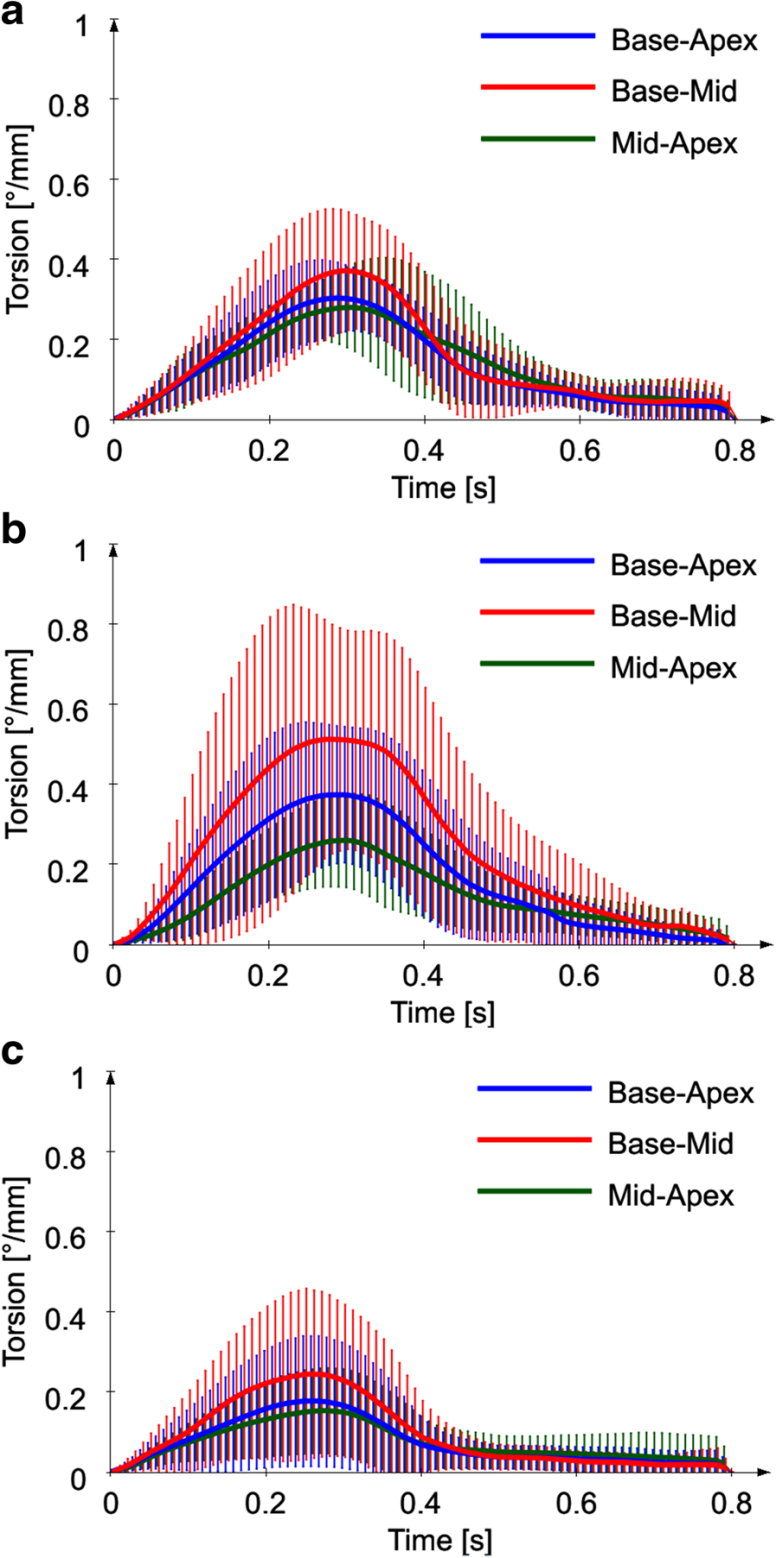
Average base-apex (blue), base-mid (red), and mid-apex (green) torsion curves as functions of time for volunteers (**a**), patients with preserved (**b**), and patients with reduced EF (**c**)

The myocardial torsion was computed for the basal vs. apical slice as a global parameter for myocardial twists of the entire LV. However, myocardial torsion can be defined between any two short-axis slices. Figure 8 shows the myocardial torsion as a function of time for base-apex (blue), base-mid (red), and mid-apex (green) pair of slices. Similarly, the subgroups of volunteers (A), patients with preserved (B) and patients with reduced (C) EF were considered separately. The maximum torsion was the highest for the base-mid slices for all three subgroups. However, the error bars in Fig. 8 show that the base-apex torsion has the lowest variability of the three.

### Inter-observer variability, reproducibility

**Fig. 9:**
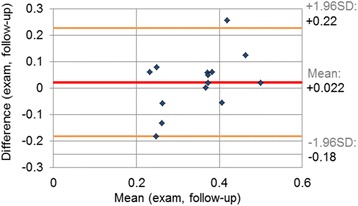
Test-retest: Difference in *T*_*max*_ obtained from two TPM datasets acquired in a two-week interval for the group of 14 volunteers

In our scan-rescan TPM study for the 14 volunteers, we observed a mean difference of +0.022 ± 0.1°/mm in the maximum torsion parameter, *T*_*max*_, as shown in Fig. 9. The relative error in the *T*_*max*_ parameter obtained in the two consecutive examinations was 25.9 %.

**Fig. 10:**
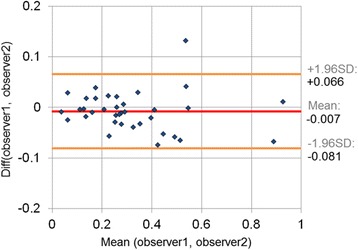
Inter-observer variability: Difference in *T*_*max*_ obtained for two observers based on the analysis of the same TPM dataset for all subjects

When comparing the *T*_*max*_ parameter obtained by two independent observers for all patients, Bland-Altman analysis revealed a mean difference of −0.007 ± 0.037°/mm and a mean relative error of 10.7 %, as shown in Fig. 10.

## Discussion

The findings of this study indicated that TPM is a promising tool to quantify LV torsion as a functional marker of cardiac disease. In this work, we investigated the maximum torsion as a potential biomarker for global myocardial twist motion in a cohort of patients with non-ischemic cardiomyopathy and healthy volunteers. The proposed torsion parameter is different from local shear measures, as it tries to globally characterize the twisting motion of the myocardium.

Firstly, the positive correlation between maximum torsion and ejection fraction showed that the maximum torsion during systole could be an additional index of global cardiac function. Moreover, torsion was also significantly lower for patients having wall motion abnormalities, even when ejection fraction is preserved, compared to healthy controls, suggesting that torsion is additionally sensitive to local changes in the myocardial motion which were not captured by ejection fraction. Secondly, when analyzing torsion transmurally, we observed that endocardial torsion was significantly higher than epicardial for normal volunteers and patients with preserved ejection fraction. Thirdly, another important finding was the positive correlation of the absolute and relative difference in torsion between the endo- and epicardial regions with ejection fraction.

Reduction in ejection fraction has been previously associated with a reduction in maximum myocardial torsion in studies performed using speckle tracking [30] and tagging CMR [7, 8, 31, 32], which was also confirmed by our results. This shows that maximum torsion can be used as an index of global cardiac function. This is also reflected by the findings in our study, namely the correlation of maximum torsion and ejection fraction, as well as the significantly lower maximum torsion for the subgroup of patients with reduced ejection fraction compared to the patients with preserved ejection fraction and to controls.

However, the added value of torsion as a cardiac parameter could be its sensitivity to local changes in myocardial motion caused by various cardiovascular diseases (hypertrophy, diastolic dysfunction) in patients with preserved ejection fraction [3]. For example, it has been inferred that if an increase in myocardial torsion is observed in early left ventricular dysfunction, it may serve as a compensatory mechanism for a reduction in left ventricular longitudinal shortening [9]. The sensitivity of torsion to manifestations of cardiovascular disease as wall motion abnormalities is also supported by the finding in our study: Maximum torsion was significantly lower in the presence of wall motion abnormalities even when ejection fraction was preserved, compared to the rest of the patients and controls. These findings indicate the potential utility of torsion to detect patients with local abnormalities despite a preserved ejection fraction. However, the number of patients with impaired wall motion and preserved ejection fraction considered in our study is too low for these results to be conclusive and larger studies are needed to confirm these findings.

Myocardial torsion can be computed between any pair of short-axis slices, taking into account their variable distance and radii. The base-mid torsion has generally higher values than the base-apex and mid-apex, especially for non-ischemic cardiomyopathy patients with preserved ejection fraction. However, the base vs. apex torsion parameter has the advantage of quantitatively describing the twisting motion of the entire left ventricle over time.

Furthermore, our results showed that myocardial torsion was higher in the endocardial tissue layer compared to the epicardial tissue layer in subjects with normal heart function. This is in accordance with the observation of a more dynamic motion pattern in the sub-endocardial fibers, which have a higher velocity than the epicardial during the cardiac cycle [13, 33]. In the literature, however, there has been some disagreement on the relative proportion of endo- and epicardial torsion depending on the imaging technique. For example, Rüssel et al. [10] used tagging in their study and found that the endocardial twist angle is larger than the epicardial, but when normalizing to the radius of the LV to obtain the torsion, the relation was inverted. This inconsistency could be a result of different methodology and calculation methods (normalization to heart size and local radii), which are not yet standardized. In contrast, using our method the relation of higher endocardial motion was preserved after normalization to the LV radius, in agreement with the study by Buchalter et al. [11] and other studies reviewed in [3].

Moreover, we examined the variation of absolute and relative difference between endo- and epicardial torsion for our patient subgroups. Our correlation analysis showed that the difference in dynamics between the endocardial and epicardial fiber clusters was significantly smaller for patients with reduced cardiac function. We observed a reduction in the maximum torsion difference between the endo- and epicardial layers, both in absolute and relative values, for progressively higher LV dysfunction. More specifically, the transmural difference in torsion was reduced close to zero for patients with ejection fraction smaller than 40 %.

The difference in torsion between endo- and epicardial layers was also associated with age. As the myocardial microfibers continue to grow throughout an adult’s life, the helical architecture becomes more elaborate. Evidence of an increase in fiber twist due to this remodeling has also been reported with other methods. Omar et al. [9] reported an increase in epicardial twist with age. In contrast, Notomi et al. [34], using Doppler imaging, reported that even though the torsion angle increases with age, when normalized by the length of the left ventricle, torsion was actually higher in younger subjects. In our study we found a significant positive association between maximum torsion and age, both globally for the entire myocardium and for each of the endocardial and epicardial bands. Also, the significant correlation detected between age and transmural torsion difference suggested that the difference between endo- and epicardial torsion is proportional to age, which could be another effect of the microfiber remodeling. However, we were not able to confirm the correlation using only the small subgroup of volunteers, to verify whether the correlation is not caused by the presence of other age-related diseases. In the future, this will be investigated this using a larger number of healthy subjects.

### Limitations

Our present study only included patients with reduced ejection fraction or wall motion abnormalities, without distinguishing between the causes for the dysfunction. As a first step, our goal was to test our method for quantifying myocardial torsion based on TPM and its capability to distinguish between patients with normal and abnormal cardiac function, as well as to explore the potential of quantifying the relation between endocardial and epicardial torsion within these groups. However, the heterogeneity of the patient cohort is a significant limitation of the work. In the future, we plan to conduct more targeted studies using more specifically selected patient groups.

In our study cohort, we did not find a significant change of torsion in the presence of late gadolinium enhancement, though such findings were previously reported (using tagging on humans [35] and animal models [36]). However, we have not taken into account the size and position of the myocardial regions showing late gadolinium enhancement, mainly because our cohort included patients with non-ischemic cardiomyopathy where the enhancement, if present, is usually more diffuse. Moreover, since our torsion parameter is global, it is possible that high myocardial velocities in the healthy regions compensate for the reduced motion in the regions with late gadolinium enhancement for patients where the ejection fraction is not substantially reduced. In the future, we plan to extend our investigation to include ischemia patients, but this time use a local torsion measure that could be correlated with the presence of local ischemia.

One drawback of the TPM CMR acquisition method used in this study is the relatively long breath-hold time required. Some of the patients in our cohort had signs of dyspnea, which can lead to incomplete breath holding during the acquisition and thus imperfect image quality. Nevertheless, all TPM data could successfully be analyzed to determine torsion and transmural torsion difference. Also, there is tradeoff between the temporal resolution that can be acquired and the required scan and breath-holding time. Overcoming such limitations and imaging myocardial velocities with a higher temporal resolution could better characterize the complex wall motion pattern.

Another limitation is the relatively high sensitivity of our measurement to the myocardium segmentation. Especially in apical slices, the lack of contrast between blood pool and myocardium in the magnitude image combined with the low velocities in the phase images makes the myocardium segmentation very challenging, even as a manual task. As a consequence, we observed outliers in the Bland-Altman analysis, which led to a moderate overall reproducibility of our method. Furthermore, in case of the outliers, the slightly different orientations of the short-axis plane positioning between scan and rescan also accounts for the larger variations. Variability in the apex segmentation could also be a reason why the torsion parameters showed relatively high variability even among healthy subjects, but further investigation is required to establish the normal limits of these variations. Therefore we think that the myocardium segmentation used to analyze velocity images should be subjected to a higher standard of precision and results from automatic and semi-automatic segmentation method should be carefully monitored corrected for optimal results.

As the thickening of the myocardium during systole is more substantial in the endocardial region compared to the epicardial, another limitation of the segmentation approach is always considering the centerline to separate between the epicardial and endocardial regions of the myocardium. In the future we will track the myocardium centerline with a similar approach which was used to propagate the manual myocardium contours from a single time point to the entire image series.

## Conclusion

TPM-based quantification of myocardial torsion was employed to derive information about changes in cardiac motion induced by non-ischemic cardiomyopathy. The maximum LV torsion could be used to detect significant differences between patients with preserved or reduced global cardiac function and was sensitive to wall-motion abnormalities, thus showing potential as a biomarker for cardiovascular disease. We have also gained new inside in the difference in torsion between the endocardial and epicardial layers and their relationship to global cardiac function. Further TPM studies are planned to investigate the variation of myocardial torsion with specific cardiovascular diseases, and the possibility of using torsion-related parameters to detect early onset or severity of cardiac diseases.

## Abbreviations

CMR: cardiovascular magnetic resonance imaging
CMR: cardiovascular magnetic resonance
ECG: electrocardiogram
EF: ejection fraction
EF: patient subgroup with reduced ejection fraction
EF+: patient subgroup with preserved ejection fraction
LGE: late gadolinium enhancement
LGE: patient subgroup without late gadolinium enhancement present
LGE+: patient subgroup with late gadolinium enhancement present
LV: left ventricle
SD: standard deviation
T: torsion
TPM: tissue phase mapping
WM: patient subgroup without wall motion abnormalities
WM+: patient subgroup with wall motion abnormalities
